# The Actual Role of CPET in Predicting Postoperative Morbidity and Mortality of Patients Undergoing Pneumonectomy

**DOI:** 10.3390/jpm15040136

**Published:** 2025-03-31

**Authors:** Antonio Mazzella, Riccardo Orlandi, Patrick Maisonneuve, Clarissa Uslenghi, Matteo Chiari, Monica Casiraghi, Luca Bertolaccini, Giovanni Caffarena, Lorenzo Spaggiari

**Affiliations:** 1 Division of Thoracic Surgery, IEO European Institute of Oncology, IRCCS, 20141 Milan, Italy; riccardo.orlandi@unimi.it (R.O.); clarissa.uslenghi@unimi.it (C.U.); matteo.chiari@ieo.it (M.C.); monica.casiraghi@ieo.it (M.C.); luca.bertolaccini@gmail.com (L.B.); giovanni.caffarena@ieo.it (G.C.); lorenzo.spaggiari@ieo.it (L.S.); 2 Division of Epidemiology and Biostatistics, IEO European Institute of Oncology, IRCCS, 20141 Milan, Italy; patrick.maisonneuve@ieo.it; 3 Department of Oncology and Haemato-Oncology, University of Milan, 20122 Milan, Italy

**Keywords:** pneumonectomy, lung function, lung cancer, CPET, VO2max, ppoVO2 max

## Abstract

This study aims to determine whether maximal oxygen consumption (VO2max) or predicted postoperative (ppo)-VO2max could still reliably predict postoperative complications and deaths in lung cancer patients undergoing pneumonectomy and which values could be more reliably considered as the optimal threshold. **Methods**: We retrospectively collected data of consecutive patients undergoing pneumonectomy for primary lung cancer at the European Oncological Institute (April 2019–April 2023). Routine preoperative assessment included cardiopulmonary exercise testing (CPET) and a lung perfusion scan. We evaluated the morbidity and mortality rates; associations between morbidity, mortality, VO2max, and ppoVO2max values were investigated through ANOVA or Fisher’s exact test as appropriate. Receiver operating characteristic (ROC) curves were applied to further explore the relation between VO2max, ppoVO2max values, and 90-day mortality. **Results**: The cardiopulmonary morbidity rate was 32.2%; the 30-day and 90-day mortality rates were 2.2% and 6.7%. The PpoVO2max values were significantly lower in patients experiencing cardiopulmonary complications or deaths compared to the whole cohort, whereas VO2max, though showing a trend towards lower values, did not reach statistical significance. A VO2max value threshold of 15 mL/kg/min correlated significantly with 90-day mortality, while a ppoVO2max cut-off of 10 mL/kg/min was significantly associated with cardiopulmonary complications and 30-day and 90-day mortality rates. ROC curve analysis revealed ppoVO2max as a better predictor of 90-day mortality compared to VO2max. **Conclusions**: CPET and a lung perfusion scan are two key elements for the preoperative evaluation of patients undergoing pneumonectomy, since it provides a holistic assessment of cardiopulmonary functionality. We recommend the routine calculation of ppoVO2max, particularly when adopting a 10 mL/kg/min threshold.

## 1. Introduction

Despite technical advances and increasing appreciation that parenchyma-sparing operations (i.e., bronchial or vascular sleeve) can cure lung cancer, in some cases, pneumonectomy remains the only operation that offers potential cure for some patients. However, the risk of complications [1,2,3] and death [4,5,6,7] after pneumonectomy is significantly higher (5–9%) than the risk of lesser resections. This depends on the abrupt removal of significant lung parenchyma, leading to respiratory, cardiocirculatory, and systemic consequences: altered exercise tolerance, deterioration of pulmonary function, increased pulmonary artery pressure, and overloaded right ventricle [8].

Thus, selecting the most appropriate candidates to pneumonectomy is of paramount importance. The selection process relies on a cardiac assessment, pulmonary function testing (PFT), cardiopulmonary exercise testing (CPET) and lung perfusion scanning [9,10,11,12,13]. Maximal oxygen consumption (VO2max) is described as the single most significant variable for risk stratification, VO2max > 20 mL/kg/min likely assuring low-risk and VO2max < 10 mL/kg/min generally contraindicating pneumonectomy [9,10,11,12,13]. However, 10 mL/kg/min < VO2max < 20 mL/kg/min indicates a heterogeneous cohort of patients at a variably increased risk of complications [9,10,11,12,13], and international guidelines do not provide separate recommendations concerning pneumonectomy, which, in addition, are based on outdated data [9,10,12].

The aim of this study was to explore whether VO2max accurately predicts the risk of pneumonectomy and to assess the value of measuring the predicted postoperative (ppoVO2)max.

## 2. Materials and Methods

### 2.1. Study Design and Population

This is a retrospective, observational study evaluating consecutive patients undergoing pneumonectomy for primary lung cancer at the European Oncological Institute (IEO) of Milan, Italy, from April 2019 to April 2023. We included all patients who were preoperatively assessed through CPET, lung perfusion scan, and depth cardio-functional exams. We excluded patients who underwent salvage or completion pneumonectomy and without a preoperative complete cardio-respiratory panel. According to institutional protocols, each patient scheduled for pneumonectomy should undergo CPET and a lung perfusion scan, starting in 2019. Preoperative, intraoperative, and postoperative data were prospectively collected and retrospectively reviewed from medical charts and surgical records. The study was conducted in accordance with the Declaration of Helsinki and reported in accordance with the STROBE guideline. The study was approved by the European Institute of Oncology Ethic Committee (UID 4430). Written informed consent was obtained from each patient.

### 2.2. Pre-Admission Exams

All patients enrolled were preoperatively assessed through physical examination, routine blood tests, cardiac ultrasound, pulmonary function testing (FEV1/DLCO), CPET, and lung perfusion scintigraphy 99Tecnetium (99Tc)-labeled macroaggregate albumin (MAA).

### 2.3. Cardiopulmonary Exercise Testing/Lung Perfusion Scan

Within 30 days before surgery, patients underwent symptom-limited CPET using a bicycle ergometer, with breath-by-breath gas exchange analysis performed with a respiratory analyzer medical system. An incremental protocol was employed, with the ramp pattern rate individually tailored based on resting functional data and expected exercise tolerance, aiming for exhaustion between 8 and 12 min [10]. All cardiac and respiratory parameters were continuously monitored. Perfusion pulmonary scintigraphy with 99Tc-MAA was employed to calculate predictive postoperative (ppo)-FEV1, ppoDLCO, and ppoVO2max, according to the method pioneered by Markos and colleagues [13], with the following formula: ppo-function = preoperative function X (1–fractional perfusion of the lung to be resected).

### 2.4. Surgical Procedure and Postoperative Course

Pneumonectomies were performed by board-certified thoracic surgeons through lateral muscle-sparing thoracotomy, and the stump was always covered, as presented before [3]. Postoperative management was already described in our previous paper [3,6]. Chest drain was removed on the 7th postoperative day, after excluding suspect bronchopleural fistula by bronchoscopy, and the patient discharged the day after, unless contraindications. All patients were reevaluated 1, 3, and 6 months after discharge on an outpatient basis. Morbidity was defined as the occurrence of grade II or more Clavien-Dindo [14] cardiopulmonary complications within 30 days from surgery, in accordance with the STS/ESTS joint statement [15]. Mortality was defined as in-hospital death or death within 30 days after surgery.

### 2.5. Statistical Analysis

The results are expressed as the percentage for qualitative parameters. For quantitative variables, the normality of the distribution was evaluated using the Shapiro–Wilk test (or Kolmogorov–Smirnov test as appropriate). The results were reported as the mean ± standard deviation (SD) for normally distributed variables or as the median and interquartile range (IQR) for variables that did not follow a normal distribution. The cut-offs for VO2max and ppoVO2max were validated using receiver operating curve (ROC) analysis. We calculated the correlation between these cut-offs and the postoperative early outcomes. *p*-values are based on ANOVA or normally distributed continuous variables, the Kruskal–Wallis test for non-normally distributed continuous variables, the Mantel–Haenszel chi-square test for trend for the ordinal variables, or Fisher’s exact test for the categorical variables. *p*-values were two-sided, and those <0.05 were considered significant. All analyses were performed with SAS software (version 9.4, Cary, NC, USA).

## 3. Results

Thus, 100 patients underwent pneumonectomy in the period April 2019–April 2023, and 90 patients met the inclusion criteria and were enrolled in the study. Patients’ demographic and baseline characteristics are reported in Table 1, stratified and compared according to the VO2max and ppoVO2max values. In the study, 29 patients (32.2%) were female, and the median age was 67 years (25–82 years). The median BMI was 24.22 (15.22–36.90). The histology of the resected lung cancers was as follows: 46 adenocarcinomas, 33 squamous cancers, 5 carcinoids, 4 undifferentiated cancers, 1 large cell neuroendocrine cancer, and 1 adenosquamous cancer. The median tumor size was 55 mm (10–180 mm), and 32 patients underwent neoadjuvant treatment.

The mean VO2max was 19.5 ± 4.3 mL/kg/min, whereas the mean ppoVO2max was 12.5 ± 3.3 mL/kg/min. Both the VO2max and ppoVO2max values were significantly lower in obese and overweight patients, higher ASA scores (subjective assessment of a patient’s overall health that is based on five classes), higher Charlson comorbidity index, and in patients affected by more comorbidities (Table 1). The association between ASA score, VO2max values, and postoperative mortality is reported in Table 2. A higher ASA score was significantly associated with a lower ppoVO2max, whereas the same relation was not found with the VO2max values and mortality.

Postoperative morbidity and mortality are reported in Table 3, and 42 patients (46.7%) developed at least one complication, 11 patients (12.2%) developed pulmonary complications, and 18 patients (20%) developed cardiac complications. The 30-day mortality was 2.2% (2 patients); the 90-day mortality was 6.7% (6 patients).

A VO2max value of 15 mL/kg/min was significantly correlated with the 90-day mortality rate (*p*: 0.02). On the other hand, the cut-off of 10 mL/kg/min in ppoVO2max was significantly associated with ARDS (*p*: 0.0007), cardiac (*p*: 0.005), and pulmonary (*p*: 0.0005) complications and 30-day (*p*: 0.03) and 90-day (*p*: 0.01) mortality rates.

**Figure 1:**
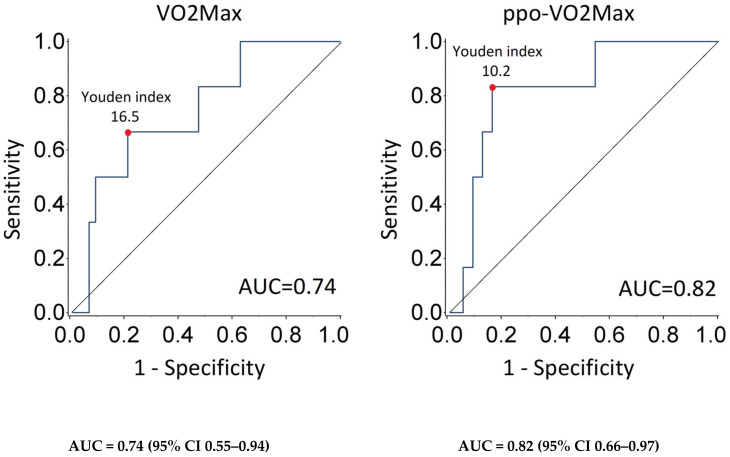
ROC curve analysis for VO2Max and ppoVO2Max as indicator of 90-day mortality.

ROC curve analysis for VO2max and ppoVO2max as predictors for 90-day mortality is reported in Figure 1. The area under the ROC curve for VO2max was 0.74 (95% CI 0.55–0.94), and the Youden’s index was 16.5. On the other hand, the AUC for ppoVO2max was 0.82 (95% CI 0.66–0.97), and the Youden’s index was 10.2, meaning a better predictivity compared to VO2max.

## 4. Discussion

Most complications and deaths after pneumonectomy fall within the cardiorespiratory category [1,7,16]. CPET provides a holistic assessment of the patient’s physiologic status; indeed, VO2max depends on several factors: respiratory, cardiovascular, musculoskeletal, circulatory, training, and effort [9,17]. Because CPET challenges the entire cardiopulmonary and oxygen delivery system under monitoring, it assesses the cardiopulmonary status and function under stress, offering a reliable estimate of the cardiopulmonary reserve. Since surgery and the perioperative phase impose significant stress on both circulatory and respiratory reserves, a preoperative exercise evaluation should be considered as a predictor of postoperative morbidity and mortality [18].

In the literature, VO2max below 15 mL/kg/min is classically associated with an increased risk of postoperative mortality after lung resection [19,20,21,22]. Nonetheless, it has been demonstrated that patients with postoperative complications after lung resection had VO2max decreasing by 3 mL/kg/min compared to those without complications [23]. Brunelli and colleagues [24] evaluated 285 patients undergoing lung resection preoperatively assessed by CPET and found that a VO2max of 12 mL/kg/min was the optimal threshold for predicting complications and mortality (33% and 13%, respectively). In this study, the authors analyzed all lung resections, including only 27 pneumonectomies, but that cut-off could not be adopted in pneumonectomies, since it is too low. Within our cohort, only four patients had a lower value. In our study, 15 mL/kg/min was the best threshold for predicting the 90-day mortality rate.

The largest study selectively focused on VO2max as a predictor of complications after pneumonectomy (150 patients), from the early 2000s [16], and it was not able to identify a clear cut-off limit for safe or prohibitive surgery. They reported an average value of 21.5 mL/kg/min in uncomplicated patients and 19.9 mL/kg/min in complicated patients.

Concerning all lung resections, in 1995, Bolliger and colleagues [17] firstly proposed the segmental estimation of VO2max, named ppoVO2max, as the only parameter able to predict mortality in patients at increased risk of complications, suggesting a cut-off value of 10 mL/kg/min. After almost two decades, Brunelli and colleagues [25] deeply analyzed the role of ppoVO2max (cut-off value 10 mL/kg/min) in predicting morbidity and mortality after lung resection (26% and 8.7%, respectively), exploring whether ppoVO2max could be precise in predicting the real postoperative VO2max value; this evaluation turned out to be inaccurate, likely due to the low number of pneumonectomies included (nine patients). For this reason, recommendations on postoperative risk prediction have been based on studies with limited sample sizes, with only a small proportion of pneumonectomies included [26].

We report a mortality rate of 2.2%, testifying to the improvement of perioperative care and a better selection of patients. Most recent studies have reported a 30-day mortality rate ranging from 5 to 8% and a 90-day mortality rate 1.5–2 times higher, whereas the 30-day major morbidity rate reached 30% [27]. Recently, Gooseman and colleagues [28] reported 22% cardiopulmonary morbidity and a 7.8% mortality rate in patients undergoing pneumonectomy with VO2max at 10–20 mL/kg/min (333 pts) and 30% cardiopulmonary complications and 4.2% mortality in those with VO2max > 20 mL/kg/min (119 pts). According to the ESTS database, pneumonectomy is currently burdened with a 6% in-hospital mortality rate [28].

Our study demonstrates that the VO2max value, when adopting a 15 mL/kg/min cut-off, is not able to reliably predict the risk of postoperative complications in lung cancer patients undergoing pneumonectomy, though they predict the risk of 90-day mortality. Conversely, a ppoVO2max cut-off of 10 mL/kg/min could better stratify the risk of postoperative complications and mortality, either at 30 or 90 days. PpoVO2max is a reliable predictor of postoperative complications and early mortality in lung cancer patients undergoing pneumonectomy, more than VO2max, which still has a role in predicting postoperative deaths. Therefore, analyzing the ROC curve, the AUC (area under the curve) of the VO2 max (0.744) appears acceptable; the AUC based on ppoVO2max (0.817) instead shows excellent results.

As expected, in our study, patients with a higher BMI, higher ASA score, and more comorbidities gained worse VO2max and consequently worse ppoVO2max than other counterparts, underlining the fact that the performance of CPET is influenced by several factors.

Based on our experience, each candidate for pneumonectomy should undergo a CPET evaluation and pulmonary perfusion scan, because the wide extent of planned resection automatically places patients in the highest risk group. The derived ppoVO2max allows to decide whether these patients can safely undergo surgery better than VO2max.

This study presents some limitations: first of all, its retrospective nature; secondly, the lack of a comparative group of patients not assessed by CPET; thirdly, the lack of information on patients who did not undergo surgery due to unfitting. Nonetheless, it represents one of the largest and most homogeneous series of pneumonectomies assessed by CPET, providing high reliability in the results.

## 5. Conclusions

CPET associated with a lung perfusion scan is a key element for the preoperative evaluation of patients undergoing pneumonectomy. The ppoVO2max values were significantly lower in pneumonectomy patients experiencing cardiopulmonary complications or deaths as compared to the entire group that was studied. The VO2max measurements showed the same trend, but they did not reach statistical significance. We recommend routinely calculating the ppoVO2max before pneumonectomies and adopting 10 mL/kg/min as the threshold of relative safety.

## Figures and Tables

**Table 1:** Patients’ characteristics.

	Patients	VO2max						ppo-VO2				
	N (%)	Mean ± SD	*p*-Value	<15	≥15	*p*-Value		Median (IQR)	*p*-Value	<10	≥10	*p*-Value
All	90 (100.0)	19.5 ± 4.3		11 (100.0)	79 (100.0)			12.2 (10.4–14.1)		17 (100.0)	73 (100.0)	
Age												
<60	19 (21.1)	20.7 ± 4.6		2 (18.2)	17 (21.5)			12.5 (11.7–15.1)		3 (17.6)	16 (21.9)	
60–64	17 (18.9)	20.1 ± 3.6		2 (18.2)	15 (19.0)			13.0 (11.1–14.9)		2 (11.8)	15 (20.5)	
65–69	25 (27.8)	19.4 ± 5.3		4 (36.4)	21 (26.6)			12.2 (10.2–13.1)		5 (29.4)	20 (27.4)	
70+	29 (32.2)	18.6 ± 3.6	0.36	3 (27.3)	26 (32.9)	0.84		11.7 (10.1–13.7)	0.38	7 (41.2)	22 (30.1)	0.56
Sex												
Male	61 (67.8)	20.1 ± 4.4		6 (54.5)	55 (69.6)			12.6 (10.6–14.6)		11 (64.7)	50 (68.5)	
Female	29 (32.2)	18.3 ± 4.0	0.06	5 (45.5)	24 (30.4)	0.32		11.6 (10.2–12.9)	0.08	6 (35.3)	23 (31.5)	0.78
BMI												
Normal weight	55 (61.1)	20.8 ± 4.6		3 (27.3)	52 (65.8)			12.9 (11.2–14.9)		8 (47.1)	47 (64.4)	
Over weight	30 (33.3)	17.6 ± 3.2		6 (54.5)	24 (30.4)			11.5 (9.2–12.8)		8 (47.1)	22 (30.1)	
Obese	5 (5.6)	17.3 ± 2.9	0.002	2 (18.2)	3 (3.8)	0.008		12.2 (10.4–13.4)	0.038	1 (5.9)	4 (5.5)	0.23
ASA score												
1	2 (2.2)	29.3 ± 4.2		0 (0.0)	2 (2.5)			18.5 (15.1–21.9)		0 (0.0)	2 (2.7)	
2	55 (61.1)	20.7 ± 4.0		5 (45.5)	50 (63.3)			13.0 (11.5–14.8)		5 (29.4)	50 (68.5)	
3	33 (36.7)	17.1 ± 3.1	<0.0001	6 (54.5)	27 (34.2)	0.17		11.0 (9.7–12.5)	0.0002	12 (70.6)	21 (28.8)	0.002
CCI												
Mild	6 (6.7)	24.2 ± 5.8		1 (9.1)	5 (6.3)			17.0 (11.8–20.9)		1 (5.9)	5 (6.8)	
Moderate	35 (38.9)	20.3 ± 4.1		3 (27.3)	32 (40.5)			12.4 (11.5–14.5)		5 (29.4)	30 (41.1)	
Severe	49 (54.4)	18.5 ± 3.9	0.004	7 (63.6)	42 (53.2)	0.61		11.7 (10.2–13.6)	0.039	11 (64.7)	38 (52.1)	0.38
Comorbidity												
No	39 (43.3)	21.6 ± 4.0		1 (9.1)	38 (48.1)			13.3 (11.5–14.9)		4 (23.5)	35 (47.9)	
Yes	51 (56.7)	18.0 ± 3.9	<0.0001	10 (90.9)	41 (51.9)	0.02		11.7 (9.9–13.1)	0.005	13 (76.5)	38 (52.1)	0.10
Cardiovascular												
No	60 (66.7)	20.7 ± 4.1		4 (36.4)	56 (70.9)			12.9 (11.1–14.8)		8 (47.1)	52 (71.2)	
Yes	30 (33.3)	17.2 ± 3.8	0.0002	7 (63.6)	23 (29.1)	0.04		11.1 (9.9–12.6)	0.001	9 (52.9)	21 (28.8)	0.09
Pulmonary												
No	81 (90.0)	19.8 ± 4.3		9 (81.8)	72 (91.1)			12.3 (10.6–14.2)		13 (76.5)	68 (93.2)	
Yes	9 (10.0)	17.1 ± 4.0	0.07	2 (18.2)	7 (8.9)	0.30		10.2 (9.2–12.5)	0.09	4 (23.5)	5 (6.8)	0.06
Previous malignancy												
No	74 (82.2)	20.0 ± 4.2		7 (63.6)	67 (84.8)			12.3 (11.0–14.2)		13 (76.5)	61 (83.6)	
Yes	16 (17.8)	17.6 ± 4.6	0.047	4 (36.4)	12 (15.2)	0.10		10.6 (9.8–14.0)	0.30	4 (23.5)	12 (16.4)	0.49
Smoking												
No	27 (30.0)	20.1 ± 4.5		3 (27.3)	24 (30.4)			12.5 (10.8–16.0)		3 (17.6)	24 (32.9)	
Current	24 (26.7)	20.4 ± 4.3		2 (18.2)	22 (27.8)			12.3 (11.0–13.7)		3 (17.6)	21 (28.8)	
Ex	37 (41.1)	18.4 ± 4.1	0.15	6 (54.5)	31 (39.2)	0.72		11.7 (9.7–13.6)	0.16	11 (64.7)	26 (35.6)	0.13
COPD												
No	73 (81.1)	19.7 ± 4.4		8 (72.7)	65 (82.3)			12.2 (10.6–13.9)		10 (58.8)	63 (86.3)	
Yes	17 (18.9)	18.9 ± 4.2	0.50	3 (27.3)	14 (17.7)	0.43		12.2 (9.2–14.2)	0.46	7 (41.2)	10 (13.7)	0.02
Diabetes												
No	82 (91.1)	19.6 ± 4.3		10 (90.9)	72 (91.1)			12.2 (10.4–14.2)		16 (94.1)	66 (90.4)	
Yes	8 (8.9)	18.5 ± 4.7	0.50	1 (9.1)	7 (8.9)	1.00		12.5 (11.4–13.0)	0.76	1 (5.9)	7 (9.6)	1.00
Cardiac (Hypertension)												
No	55 (61.1)	20.5 ± 4.3		4 (36.4)	51 (64.6)			12.5 (11.0–14.9)		6 (35.3)	49 (67.1)	
Yes	35 (38.9)	18.0 ± 4.0	0.006	7 (63.6)	28 (35.4)	0.10		11.7 (9.2–13.1)	0.014	11 (64.7)	24 (32.9)	0.03

Data are missing for some variables: smoking (*n* = 2). IQR: interquartile range. *ppo*–VO2 = VO2max × (100 − Perfusion)/100. *p*-values are based on ANOVA for normally distributed continuous variables, the Kruskal–Wallis test for non-normally distributed continuous variables, the Mantel–Haenszel chi-square test for trend for ordinal variables, or Fisher’s exact test for categorical variables.

**Table 2:** Association between ASA score, VO2max, ppo-VO2, and mortality at 30 and 90 days.

	Patients	VO2max		ppo-VO2		Mortality	
		<15	≥15	<10	≥10	30 days	90 days
	N (%)	N (%)	N (%)	N (%)	N (%)	N (%)	N (%)
All patients	90 (100)	12 (12.2)	78 (87.8)	17 (18.9)	73 (81.1)	2 (2.2)	6 (6.7)
ASA score							
I	2 (2.2)	0 (0.0)	2 (100.)	0 (0.0)	2 (100.)	0 (0.0)	0 (0.0)
II	55 (61.1)	5 (9.1)	50 (90.1)	5 (9.1)	50 (90.1)	1 (1.8)	3 (5.5)
III	33 (36.7)	6 (18.2)	27 (81.8)	12 (36.4)	21 (63.6)	1 (3.0)	3 (9.1)
*p*-value		**0.17**		**0.002**		**0.67**	**0.45**

ppo-VO2: VO2max × (100 − perfusion)/100. *p*-values are based on the Mantel–Haenszel chi-square test for the trend.

**Table 3:** Postoperative and early outcomes.

	Patients	VO2max						ppo-VO2				
	N (%)	Mean ± SD	*p*-Value	<15	≥15	*p*-Value		Median (IQR)	*p*-Value	<10	≥10	*p*-Value
All	90 (100.0)	19.5 ± 4.3		11	79			12.2 (10.4–14.1)		17 (100)	73 (100)	
												
ARDS	6 (6.7)	16.6 ± 3.1	0.09	2 (18.2)	4 (5.1)	0.16		9.5 (8.8–9.9)	**0.007**	5 (29.4)	1 (1.4)	**0.0007**
Fistula	9 (10.0)	22.1 ± 5.3	0.06	0 (0.0)	9 (11.4)	0.59		13.8 (12.5–14.9)	0.09	0 (0.0)	9 (12.3)	0.20
Any complications	42 (46.7)	19.4 ± 4.7	0.74	7 (63.6)	35 (44.3)	0.34		12.2 (9.9–13.8)	0.40	12 (70.6)	30 (41.1)	**0.03**
Pulmonary	11 (12.2)	17.3 ± 4.6	0.07	3 (27.3)	8 (10.1)	0.13		9.9 (8.7–12.5)	**0.008**	7 (41.2)	4 (5.5)	**0.0005**
Cardiac	18 (20.0)	18.2 ± 4.7	0.15	3 (27.3)	15 (19.0)	0.69		10.7 (9.2–12.6)	**0.008**	8 (47.1)	10 (13.7)	**0.005**
Other	32 (35.6)	19.4 ± 4.3	0.85	4 (36.4)	28 (35.4)	1.00		12.3 (10.5–14.3)	0.81	7 (41.2)	25 (34.3)	0.59
Mortality 30 days	2 (2.2)	17.5 ± 5.3	0.49	1 (9.1)	1 (1.3)	0.23		9.3 (8.7–9.9)	0.31	2 (11.8)	0 (0.0)	**0.03**
Mortality 90 days	6 (6.7)	16.5 ± 3.1	0.07	3 (27.3)	3 (3.8)	**0.02**		9.5 (9.0–10.2)	0.055	4 (23.5)	2 (2.7)	**0.01**

ppo-VO2: VO2max × (100 − perfusion)/100. IQR: interquartile range. *p*-values are based on ANOVA for normally distributed continuous variables, the Kruskal–Wallis test for non-normally distributed continuous variables, or Fisher’s exact test for categorical variables.

## Data Availability

No new data were created.

## Abbreviations

PFT	pulmonary function testing
CPET	cardiopulmonary exercise testing
99Tc-MAA	99Tecnetium (99Tc)-labeled macroaggregate albumin (MAA)
VO2max	maximal oxygen consumption
(ppoVO2)VO2max	predictive postoperative maximal oxygen consumption
FEV1	expiratory forced volume
DLCO	diffusing capacity of the lungs for carbon monoxide
